# Short and long-term effect of dexamethasone on the transcriptome profile of primary human trabecular meshwork cells in vitro

**DOI:** 10.1038/s41598-022-12443-7

**Published:** 2022-05-18

**Authors:** Kandasamy Kathirvel, Karen Lester, Ravinarayanan Haribalaganesh, Ramasamy Krishnadas, Veerappan Muthukkaruppan, Brian Lane, David A. Simpson, Kasia Goljanek-Whysall, Carl Sheridan, Devarajan Bharanidharan, Colin E. Willoughby, Srinivasan Senthilkumari

**Affiliations:** 1 Department of Ocular Pharmacology, Aravind Medical Research Foundation, #1, Anna Nagar, Madurai, Tamil Nadu 625020 India; 2 Genomic Medicine, Biomedical Sciences Research Institute, Ulster University, Coleraine, Northern Ireland, UK; 3 Glaucoma Clinic, Aravind Eye Hospital, Madurai, Tamil Nadu India; 4 Aravind Medical Research Foundation, Madurai, Tamil Nadu India; 5 Translational Radiobiology Group, Division of Cancer Sciences, Manchester Academic Health Science Centre, Christie NHS Foundation Trust Hospital, University of Manchester, Manchester, M20 4BX UK; 6 Institute of Life Course and Medical Sciences, University of Liverpool, Liverpool, L7 8TX UK; 7 School of Medicine, Dentistry and Biomedical Sciences, The Wellcome – Wolfson Institute for Experimental Medicine, Queen’s University Belfast, Belfast, UK; 8 School of Medicine, Physiology, National University of Ireland Galway, Galway, H91 W5P7 Ireland; 9 Department of Eye and Vision Science, Institute of Life Course and Medical Sciences, University of Liverpool, Liverpool, L7 8TX UK; 10 Department of Bioinformatics, Aravind Medical Research Foundation, Madurai, Tamil Nadu India

**Keywords:** Cell biology, Molecular biology, Medical research

## Abstract

In the quest of identifying newer molecular targets for the management of glucocorticoid-induced ocular hypertension (GC-OHT) and glaucoma (GCG), several microarray studies have attempted to investigate the genome-wide transcriptome profiling of primary human trabecular meshwork (TM) cells in response to dexamethasone (DEX). However, no studies are reported so far to demonstrate the temporal changes in the expression of genes in the cultured human TM cells in response to DEX treatment. Therefore, in the present study, the time-dependent changes in the genome-wide expression of genes in primary human TM cells after short (16 hours: 16 h) and long exposure (7 days: 7 d) of DEX was investigated using RNA sequencing. There were 199 (118 up-regulated; 81 down-regulated) and 525 (119 up-regulated; 406 down-regulated) DEGs in 16 h and 7 d treatment groups respectively. The unique genes identified in 16 h and 7 d treatment groups were 152 and 478 respectively. This study found a distinct gene signature and pathways between two treatment regimes. Longer exposure of DEX treatment showed a dys-regulation of Wnt and Rap1 signaling and so highlighted potential therapeutic targets for pharmacological management of GC-OHT/glaucoma.

## Introduction

Glucocorticoids (GCs) use is widespread in ophthalmology and continues to be the mainstay of treatment for inflammatory eye diseases^1^. The prolonged use of GCs elevates intraocular pressure (IOP) in susceptible individuals^2^. It is reported that the increase in IOP happens in 40% of the individuals without glaucoma (steroid responder) and in up to 90% of primary open angle glaucoma^2–4^. The risk of IOP elevation, and the time-frame during which this occurs, is dependent on steroid potency, pharmacokinetics, duration of treatment, routes of administration, as well as individual differences in responsiveness^5^. The exact molecular mechanism for GC-induced elevated IOP is not clearly understood.

In the trabecular meshwork (TM), dexamethasone (DEX) is known to induce cell remodeling^5–9^ and extracellular matrix (ECM) remodeling which are implicated in ocular hypertension caused by GCs^10^. Several studies have examined global changes in gene expression in the cultured human TM cells treated with dexamethasone (DEX) using cDNA or oligonucleotide arrays^9,11–20^. However, the overall findings were not consistent across studies because of the different duration of treatment (varied from 24 h to 21 days) and different microarray platforms used in these studies. These studies were restricted due to a single/ limited time point.

Since the responses of the TM to DEX treatment are dose and duration dependent, no studies have been reported so far to demonstrate the temporal changes in the expression of genes in the cultured human TM cells. Therefore, in the present study, the time-dependent changes in the genome-wide expression of genes in primary human TM cells after short (16 hours: 16 h) and long exposure (7 days: 7 d) of 100 nM DEX were assessed using RNA-Sequencing (RNA-Seq). This is the first study report on the temporal changes in the expression of genes in the cultured human TM cells after two different DEX exposure time points. We hypothesized that the shorter DEX exposure may reveal acute and initiating alterations in gene expression which is responsible for the sustained ocular hypertensive phenotypes in the TM which persists with longer exposure of DEX. The results of the present study revealed a distinct gene signature observed between two treatment groups and found that long exposure of DEX treatment showed dys-regulation of two signaling pathways such as Wnt and Rap1 signaling. These pathways could serve as potential therapeutic targets for pharmacological management of GC-OHT/glaucoma.

## Results

### Quality of RNA-seq data

An average of 35 million reads were generated from each sample of 16 h treated group and obtained by de-multiplexing. Each base from mRNA-sequencing reads of 16 h and 7 d DEX-treated cells met the expected Phred score criteria, Q > 30.

In Principle Component Analysis, the samples got dispersed into different regions of the plot corresponding to the principal sources of variation within the experiment. Both control as well treated groups segregate on the first and second principal component axes, but there is a clear relationship between donor eyes, indicating the need to incorporate donor source as a factor in an expression analysis of this data.

### Differential gene expression analysis

**Figure 1:**
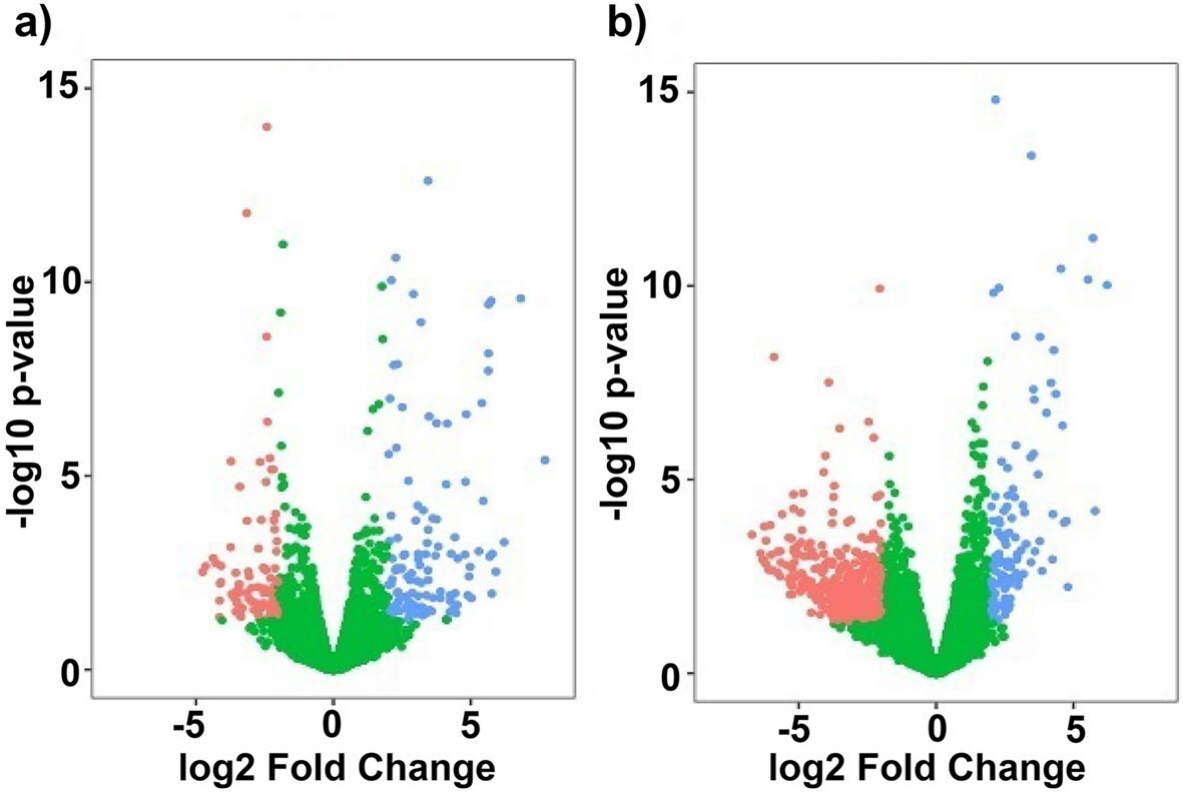
Volcano Plot Showing the Distribution of DEGs. The fold of change (log2) and *p* value (− log10) of genes from (**a**) 16 h DEX treatment (Group A), (**b**) 7 d DEX treatment (Group B) are represented.

**Figure 2:**
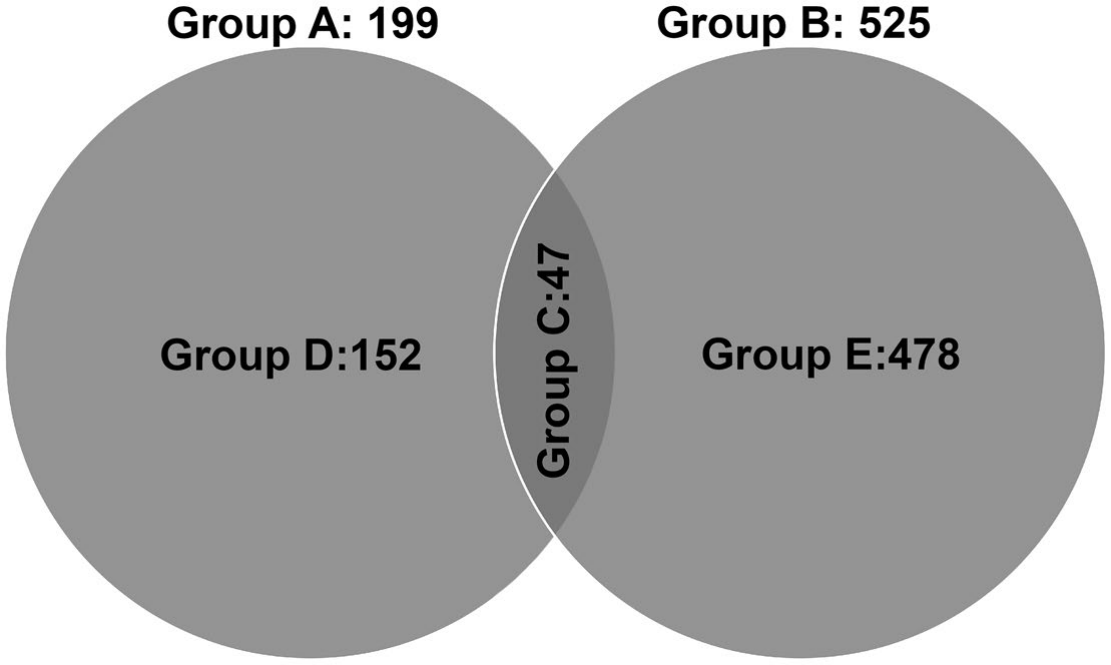
Venn diagram showing differentially expression groupings. DEGs of RNA seq data identified in different study groups are shown. Only genes with absolute fold change 2 and significant *P* value < 0.05 were included in these groupings. Group A: DEGs between DEX and ETH treated for short duration (16 h); Group B: DEGs between DEX and ETH treated for longer duration (7 d); Group C: DEGs that overlapping between Group A and Group B; Group D: Uniquely expressed DEGs of HTM cells exposed for 16 h (Group A minus Group C); Group E: Uniquely expressed DEGs of HTM cells exposed for 7 d (Group B minus Group C).

In total 16,335 and 17,499 genes with more than ten read counts were identified in 16 h (Group A) and 7 d (Group B) DEX-treated HTM cells respectively. There were 199 (118 up-regulated; 81 down-regulated) DEGs in Group A and 525 (119 up-regulated; 406 down-regulated) DEGs in Group B identified as differentially expressed with absolute fold change 2 and probability value lesser than 0.05. The relationship between statistical significance and log FC in normalized expression between the experimental groups are shown in the volcano plot (Fig. 1). The commonly expressed genes from Group C were found to be 47 and out of which ZBTB16, SAA1, OCA2, RGCC and FKBP5 were identified as the most up-regulated and KRT15, FST, and TP63 the most down-regulated genes. Unique genes identified in 16 h (Group D) and 7 d (Group E) treatment groups were 152 and 478 respectively (Fig. 2).

In Group A, the genes such as CCAT1 (FC = 5.9), CIDEC (FC = 5.7), NDST4 (FC = 5.7), FAM105A (FC = 5.6) and APOB (FC = 4.2) genes were found to be highly up-regulated, while LRRN1 (FC =  − 4.1), FGF16 (FC =  − 4.1) and SERPINB2 (FC =  − 3.7) were down-regulated and these genes were uniquely expressed in Group D whereas, in Group B, MYOC (FC = 5.7), APOD (FC = 4.7), PRODH (FC = 4.5), LSP1 (FC = 4.2) and IGF2 (FC = 3.7) genes were identified as highly up-regulated, while NPY (FC =  − 6.7), PRSS22 (FC =  − 6.3), ACPP (FC =  − 6.2) and RLN1 (FC =  − 6.1) were down-regulated genes and were found to be uniquely expressed in Group E (Supplementary Table S3a–e). Interestingly, a greater number of significantly DEGs were identified in 7 d treatment group as compared to 16 h treatment group which indicates that prolonged exposure of DEX significantly alters the gene expression profile in HTM cells.

### Pathways enrichment analysis

The pathway enrichment analysis of DEGs from each group revealed some unique significantly altered pathways (Table 1). They were clustered into several functional categories. Interestingly, Drug metabolism—cytochrome P450 and tyrosine metabolism pathways were commonly expressed as up-regulated pathways in Group A (16 h) and Group B (7 d). The up-regulation of cytokine-cytokine receptor interaction pathway was found only in 16 h group (Group D; unique 16 h) whereas Rap1 signaling pathway was observed only in 7 d group (Group E; unique 7 d). The down-regulated pathways found in Group D were hematopoietic cell lineage, neuroactive ligand-receptor interaction and calcium signaling pathway and in Group E, the pathways such as Wnt signaling pathway, pathways in cancer, cell adhesion molecules and proteoglycans in cancer pathways were found (Table 1).

**Table 1:** List of enriched up/down-regulated pathways.

Group	Pathway	Fold enrichment	P value	Genes
Group A	Tyrosine metabolism	20.7	8.3E−04	PNMT, MAOA, ADH1B, AOX1
	Cytokine-cytokine receptor interaction	3.7	4.0E−02	LEP, CCL3L3, IL1R2, IL18, IFNLR1
	Amphetamine addiction	8.2	4.9E−02	GRIA1, MAOA, PPP1R1B
	Drug metabolism—cytochrome P450	8.0	5.1E−02	MAOA, ADH1B, AOX1
	Neuroactive ligand-receptor interaction	− 5.3	4.0E−03	GRIN2A, GABRA4, TACR1, GRPR, MCHR1, NTSR1
	Cytokine-cytokine receptor interaction	− 5.1	1.4E−02	IL1A, CSF2, IL1B, TNFRSF9, CXCR4
	Calcium signaling pathway	− 5.5	3.2E−02	GRIN2A, TACR1, GRPR, NTSR1
	Salmonella infection	− 8.9	4.2E−02	IL1A, CSF2, IL1B
	Hematopoietic cell lineage	− 8.5	4.5E−02	IL1A, CSF2, IL1B
	Rheumatoid arthritis	− 8.4	4.6E−02	IL1A, CSF2, IL1B
Group B	Drug metabolism—cytochrome P450	15.2	3.6E−05	ADH4, MAOA, ADH1B, ADH1A, AOX1, FMO2
	Tyrosine metabolism	24.6	4.1E−05	ADH4, MAOA, ADH1B, ADH1A, AOX1
	Retinol metabolism	10.7	5.5E−03	ADH4, ADH1B, ADH1A, AOX1
	Fatty acid degradation	12.3	2.3E−02	ADH4, ADH1B, ADH1A
	Glycolysis/gluconeogenesis	7.7	5.5E−02	ADH4, ADH1B, ADH1A
	Chemical carcinogenesis	6.4	7.5E−02	ADH4, ADH1B, ADH1A
	Cell adhesion molecules (CAMs)	− 4.6	8.6E−06	ICAM2, SELE, SELP, HLA-DMB, CDH3, CLDN3, CDH1, PECAM1, HLA-DRA, HLA-DOA, HLA-DQA2, HLA-DQA1, HLA-DRB1, HLA-DQB1
	Allograft rejection	− 8.9	1.1E−04	HLA-DMB, HLA-DRA, HLA-DOA, HLA-DQA2, HLA-DRB1, HLA-DQA1, HLA-DQB1
	Basal cell carcinoma	− 6.9	1.3E−04	WNT10B, SHH, WNT10A, WNT7B, PTCH2, FZD10, WNT2, WNT4
	Type I diabetes mellitus	− 7.8	2.3E−04	HLA-DMB, HLA-DRA, HLA-DOA, HLA-DQA2, HLA-DRB1, HLA-DQA1, HLA-DQB1
	Inflammatory bowel disease (IBD)	− 5.8	3.8E−04	HLA-DMB, RORC, HLA-DRA, HLA-DOA, HLA-DQA2, HLA-DRB1, HLA-DQA1, HLA-DQB1
	Autoimmune thyroid disease	− 6.3	7.5E−04	HLA-DMB, HLA-DRA, HLA-DOA, HLA-DQA2, HLA-DRB1, HLA-DQA1, HLA-DQB1
	Pancreatic secretion	− 4.0	3.5E−03	CPA3, CPB1, FXYD2, KCNQ1, PLA2G2A, CLCA2, CD38, ATP1A2
	cAMP signaling pathway	− 2.6	9.2E−03	GRIA2, CHRM1, NPY, FXYD2, PDE3B, CNGA1, ADRB1, ATP1A2, SSTR1, SSTR2, GRIN1
	Hippo signaling pathway	− 2.8	1.5E−02	WNT10B, WNT10A, CDH1, WNT7B, RASSF6, FZD10, WNT2, BMP7, WNT4
	Wnt signaling pathway	− 2.7	2.7E−02	WNT10B, WNT10A, SFRP2, MMP7, WNT7B, FZD10, WNT2, WNT4
	Signaling pathways regulating pluripotency of stem cells	− 2.7	2.9E−02	WNT10B, WNT10A, WNT7B, IGF1, FZD10, WNT2, ISL1, WNT4
	Pathways in cancer	− 1.8	3.9E−02	WNT10B, WNT10A, WNT7B, PTCH2, CBLC, KLK3, FZD10, IGF1, MMP9, SHH, GNG4, CDH1, WNT2, NKX3-1, WNT4
	cGMP-PKG signaling pathway	− 2.4	5.1E−02	NOS3, FXYD2, PDE2A, PDE3B, CNGA1, ADRB1, ATP1A2, ADRA2A
	Adrenergic signaling in cardiomyocytes	− 2.4	7.3E−02	MYL4, TNNT2, FXYD2, KCNQ1, ADRB1, ATP1A2, CACNG4
	Cardiac muscle contraction	− 3.1	7.5E−02	MYL4, TNNT2, FXYD2, ATP1A2, CACNG4
Group C	Tyrosine metabolism	45.4	2E−3	MAOA, ADH1B, AOX1
	Drug metabolism—cytochrome P450	23.3	6E−3	MAOA, ADH1B, AOX1
	Tryptophan metabolism	26.5	6.8E−2	MAOA, AOX1
Group D	Cytokine-cytokine receptor interaction	4.5	5.1E−2	CCL3L3, IL1R2, IL18, IFNLR1
	Neuroactive ligand-receptor interaction	− 5.4	1.1E−2	GRIN2A, GABRA4, TACR1, GRPR, NTSR1
	Calcium signaling pathway	− 6.7	1.9E−2	GRIN2A, TACR1, GRPR, NTSR1
	Salmonella infection	− 10.8	2.8E−2	IL1A, CSF2, IL1B
	Hematopoietic cell lineage	− 10.3	3.1E−2	IL1A, CSF2, IL1B
	Rheumatoid arthritis	− 10.2	3.2E−2	IL1A, CSF2, IL1B
	Amoebiasis	− 8.5	4.4E−2	CSF2, SERPINB2, IL1B
	Measles	− 6.7	6.7E−2	IL1A, IL1B, TACR1
	Circadian rhythm	− 19.3	9.5E−2	PER3, NR1D1
Group E	Drug metabolism—cytochrome P450	10.5	3.1E−2	ADH4, ADH1A, FMO2
	Rap1 signaling pathway	4.5	5.2E−2	FPR1, KDR, FGFR4, RAPGEF5
	Cell adhesion molecules (CAMs)	− 4.8	0.000	ICAM2, SELE, SELP, HLA-DMB, CDH3, CLDN3, CDH1, PECAM1, HLA-DRA, HLA-DOA, HLA-DQA2, HLA-DQA1, HLA-DRB1, HLA-DQB1
	Type I diabetes mellitus	− 8.2	0.000	HLA-DMB, HLA-DRA, HLA-DOA, HLA-DQA2, HLA-DRB1, HLA-DQA1, HLA-DQB1
	Antigen processing and presentation	− 5.2	1E−3	CD74, HLA-DMB, HLA-DRA, HLA-DOA, HLA-DQA2, HLA-DRB1, HLA-DQA1, HLA-DQB1
	cAMP signaling pathway	− 2.7	7E-3	GRIA2, CHRM1, NPY, FXYD2, PDE3B, CNGA1, ADRB1, ATP1A2, SSTR1, SSTR2, GRIN1
	Proteoglycans in cancer	− 2.7	7E−3	WNT10B, WNT10A, HPSE2, WNT7B, CBLC, IGF1, FZD10, WNT2, HOXD10, MMP9, WNT4
	Wnt signaling pathway	− 2.8	2.1E−2	WNT10B, WNT10A, SFRP2, MMP7, WNT7B, FZD10, WNT2, WNT4
	Signaling pathways regulating pluripotency of stem cells	− 2.8	2.3E−2	WNT10B, WNT10A, WNT7B, IGF1, FZD10, WNT2, ISL1, WNT4
	Pathways in cancer	− 1.9	2.7E−2	WNT10B, WNT10A, WNT7B, PTCH2, CBLC, KLK3, FZD10, IGF1, MMP9, SHH, GNG4, CDH1, WNT2, NKX3-1, WNT4
	Phagosome	− 2.6	3.2E−2	HLA-DMB, SFTPA2, HLA-DRA, HLA-DOA, HLA-DQA2, HLA-DRB1, HLA-DQA1, HLA-DQB1
	Hippo signaling pathway	− 2.6	3.3E−2	WNT10B, WNT10A, CDH1, WNT7B, FZD10, WNT2, BMP7, WNT4
	cGMP-PKG signaling pathway	− 2.5	4.1E−2	NOS3, FXYD2, PDE2A, PDE3B, CNGA1, ADRB1, ATP1A2, ADRA2A
	Adrenergic signaling in cardiomyocytes	− 2.5	6E−2	MYL4, TNNT2, FXYD2, KCNQ1, ADRB1, ATP1A2, CACNG4
	Cardiac muscle contraction	− 3.3	6.5E−2	MYL4, TNNT2, FXYD2, ATP1A2, CACNG4
	Dilated cardiomyopathy	− 2.9	9E−2	DES, TNNT2, ADRB1, IGF1, CACNG4

Group A: DEGs between DEX and ETH treated for short duration (16 h); Group B: DEGs between DEX and ETH treated for longer duration (7 d); Group C: DEGs that overlapping between Group A and Group B; Group D: Uniquely expressed DEGs of HTM cells exposed for 16 h (Group A minus Group C); Group E: Uniquely expressed DEGs of HTM cells exposed for 7 d (Group B minus Group C).

### Validation RNA seq data by RT-PCR

**Figure 3:**
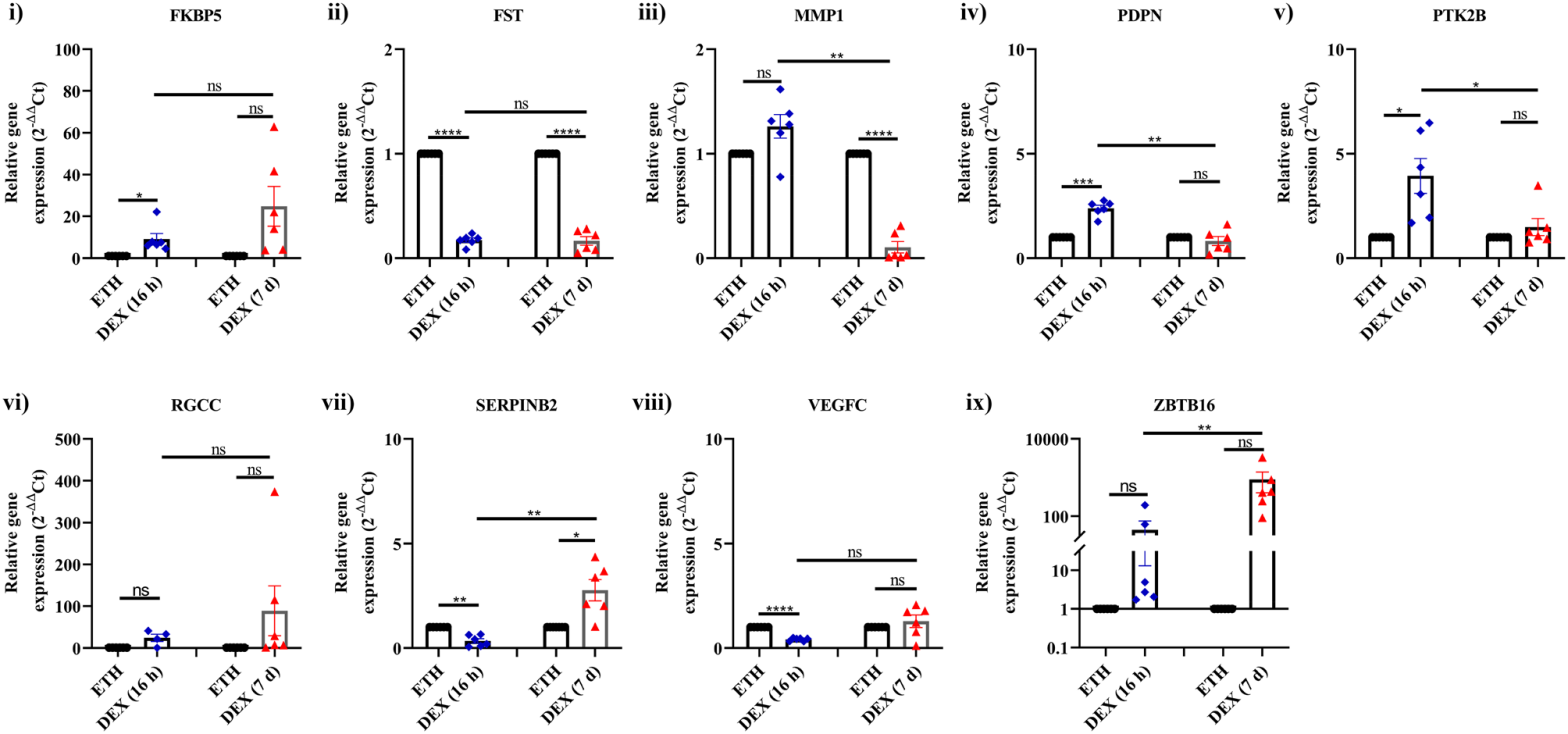
Validation of DEGs by qPCR. Expression profile of selected genes identified from RNA-seq validated by qPCR is shown. Primary HTM cells were treated with 100 nM DEX or 0.1% ETH for 16 h and 7 d. Gene expressions were normalized to GAPDH (16 h group) and ACTB (7 d group), and analyzed using the 2^−ΔΔCT^ method. The data is represented as mean ± SEM. *P < 0.01; **P < 0.005; ***P < 0.001; ****P < 0.0001. Paired 2-tailed Student’s t test.

To validate results obtained from the RNA-seq analysis, 9 candidate genes were selected for q-PCR analysis based on their statistical significance in the RNA-seq data set and on the basis of published literature providing evidence of their involvement in glaucoma pathophysiology. Eight of the nine candidate genes demonstrated consistent trends with RNA seq data (Fig. 3, Table 2). Though MMP-1 expression was found to be down-regulated in RNA-seq data, it was found to be up-regulated in qPCR analysis but the difference was not statistically significant.

**Table 2:** Comparison between RNA sequencing findings and qPCR.

S. no.	Name of the gene	Log2 fold change			
		Group A		Group B	
		RNA seq	qPCR	RNA seq	qPCR
1	FKBP5	2.91	2.97	4.47	3.94
2	FST	− 2.45	− 2.61	− 2.04	− 2.88
3	MMP1	− 1.97	0.30	− 1.47	− 4.53
4	PDPN	1.35	1.24	− 0.001	− 0.60
5	PTK2B	2.34	1.79	1.8	0.38
6	RGCC	4.79	3.54	3.59	4.48
7	SERPINB2	− 3.73	− 2.10	− 0.41	1.32
8	VEGFC	− 1.32	− 1.30	− 0.82	− 0.10
9	ZBTB16	7.70	3.19	6.47	8.90

Group A: DEGs between DEX and ETH treated 16 h; Group B: DEGs between DEX and ETH treated for 7 d.

*FKBP5* FK506 binding protein 5, *FST* follistatin, *MMP1* matrix metalloproteinase-1, *PDPN* podoplanin; protein tyrosine kinase 2 beta, *RGCC* regulator of cell cycle, *SERPINB2* serpin family B member 2, *VEGFC* vascular endothelial growth factor-C, *ZBTB16* zinc finger and BTB domain containing 16.

## Discussion

The present study utilized RNA-seq to explore genome-wide, time-dependent changes in the expression of genes in primary HTM cells in response to DEX treatment for a shorter (16 h) or longer duration (7 d) of exposure. Several previous studies identified the global changes in the expression of DEGs in response to DEX treatment in cultured HTM cells using cDNA and oligonucleotide arrays^11–20^. However, no studies are available to-date to demonstrate the temporal changes in the expression of genes in cultured HTM cells in response to DEX treatment. Therefore, in the present study two different DEX exposure time points were chosen: short duration (16 h) and long duration (7 d) of exposure. A shorter DEX exposure time may reveal acute and initiating alterations in gene expression which is responsible for the sustained ocular hypertensive phenotypes in the TM cells. The long exposure time of 7 d was chosen to investigate genes altered by chronic GC exposure to further identify the molecular changes in the HTM cells resulting in GC-induced OHT and glaucoma and also to identify novel therapeutic targets.

Our study revealed a distinct gene signature and their associated pathways that were identified for both the time points studied. Interestingly, the 7 d post DEX treatment showed a higher number of significantly altered genes (DEGs: 525–119, up-regulated and 406, down-regulated) as compared to 16 h treatment (DEGs: 199–118, up-regulated and 81, down-regulated) which indicates that prolonged exposure of DEX significantly alters the gene expression profile in HTM cells. A common number of genes that were up-regulated in the previous studies were identified in the present study in both the groups^12,13,15,17,18,21,22^. However, the expression of these genes varied in fold change with time points studied (16 h versus 7 d) which confirms that DEX-induced mRNA expression is time dependent.

The present study also revealed a novel set of genes which are not reported earlier^12,13,15,17,18,21–23^. This could be due to differences in the GC responsiveness of the human donor eyes used in the present study and in fact RNA-Seq technology offers significant benefits over previous microarray technologies in terms of offering a broader dynamic range and the sequencing coverage depth allows easier detection of rare and low-abundance transcripts.

Myocilin (MYOC), a known DEX-inducible gene was significantly altered in 7 d post-treatment group (log2FC: 5.79) as compared to 16 h group (log2FC:1.3)^21^. It has been identified as one of the four major pathogenic genes for POAG and JOAG^22^. ZBTB16 (Zinc Finger and BTB Domain Containing 16), a transcriptional repressor involved in cell cycle control and suppress the expression of several genes that regulate cell proliferation were highly expressed in both groups. However, the role of ZBTB16 in HTM cells and steroid-induced glaucoma still remains unclear^23^. The oculo-cutaneous albinism 2 (OCA2) which is prominently associated with pigmentation and acts as a determinant of brown or blue eye color was identified as one of the highly expressed genes in both short and long-term treatment groups (log2FC:6.8 (16 h); log2FC:5.7 (7 d). FK506 binding protein 5 (FKBP5), a co-chaperone of heat shock protein 90 (hsp90) which regulates GC receptor sensitivity was up-regulated at both time points. Loss of DNA methylation in FKBP5 by GC, increases FKBP5 transcription^12^.

The serum amyloid A proteins (SAA1 and SAA2), which are acute phase apo-lipoproteins reported to play an important role in inflammation, infection and tissue repair were found to be up-regulated in both time-points studied^19,20^. However, the intensity of gene expression was higher in 7 d DEX-treated group as compared to 16 h group. Moreover, another isoform of serum amyloid A protein 4, SAA4 was additionally found only in 16 h group. The Angiopoietin-like 7 (ANGPTL7) protein, a member of the ANGPTL family was one of the most up-regulated in DEX-treated HTM cells at two different time points studied. The overexpression of ANGPTL7 in human TM and aqueous humor was found to alter the expression of collagen and fibronectin, myocilin and MMP1^24,25^. Rare protein-altering variants in ANGPTL7 lower IOP and protect against glaucoma^26^.

Among the most commonly down-regulated genes identified between two groups, follistatin (FST) is known to be induced by DEX treatment in TM cells^18^. FST is a stress responsive protein exerting protective effects by neutralizing TGF-β2 signaling by antagonizing BMP and activin^27^. FST is expressed in the normal and glaucomatous TM^28,29^ and this expression is increased in a dose-dependent manner with TGF-β2 treatment^29^. In this study, FST was significantly down-regulated in both groups (log2FC: 16 h: − 2.4; 7 d: − 2.0) and it was corroborated by qPCR assay. The biological activities and underlying mechanisms for FST involvement in the TM is not understood clearly but it is proposed that FST reduces ROS production in TM cells by interacting with members of the NADPH oxidase inhibitor (NOX) family^30^. Interestingly, FST can be regulated at both transcriptional and post-transcriptional levels^31,32^, which allows an opportunity to manipulate FST expression therapeutically.

Out of 47 commonly found DEGs between two treatment groups, the expression of complement factor 7 (C7), solute carrier organic anion transporter family member 2A1 (SLCO2A1) and Ras Association Domain Family Member 6 (RASSF6) were differentially altered. For example, the C7 was up-regulated in 16 h treatment group (log2FC: 5.0) whereas in the 7 d treatment group, the expression was down-regulated (log 2FC: − 2.5). C7 is a terminal component of the complement cascade and can form a membrane attack complex with other complement components (C5b, C6, C8 and C9) which constitutes a part of the innate immune system. The expression of C7 on the cell membrane acts as a regulator of an excessive pro-inflammatory reaction. In some tumors, C7 acts as a tumor suppressor and found to show a decreased mRNA expression level correlated with an increased aggressiveness of the tumors^33^. Likewise, RASSF6 was differentially expressed in response to DEX treatment and is suppressed in human cancers and its low expression level is associated with poor prognosis^34^. However, the association of C7, RASSF6 and GC-induced glaucoma is not known and warrants further investigation.

Solute carrier organic anion transporter family member 2A1 (SLCO2A1) gene is known to encode the prostaglandin transporter PGT^35^ and is involved in the process of uptake and clearance of prostaglandins^36^. The mRNA expression of this transporter is present in a variety of human ocular tissues especially in the ciliary epithelium and choroid/RPE complex^37^. Glucocorticoids are known to inhibit the production of prostaglandins in many cell types and tissues^38^. In the TM, it has been demonstrated that the DEX treatment reduced the production of prostaglandin by 60%^39^. The present study revealed that longer exposure of DEX significantly down-regulated this transporter as compared to short exposure and hence the regulation of genes upon DEX treatment is time-dependent.

In addition to the known DEX-induced genes reported in cultured HTM cells, our study revealed some unique genes identified in each treatment group (16 h and 7 d) (Supplementary Table S3d,e). For example, plasminogen activator inhibior2 (PAI-2) or serpineB2 (SERPINB2), a serine protease inhibitor of the serpin superfamily induced during inflammatory processes and infections in many cell types such as macrophages, fibroblasts, endothelial cells and differentiating keratinocytes^40^. In the present study, it is interesting to note that SERPINB2 was significantly down-regulated in 16 h treatment group (log2FC: − 3.7) whereas in 7 d treatment group, the other form of SERPINB11was significantly down-regulated (log2FC: − 4.9). Validation of SERPINB2 decreased expression could not be achieved by q-PCR (Fig. 3). Significant glucocorticoid down-regulation of SERPINB2 expression in the TM was also reported after 6–8 days of DEX treatment^15^. SERPINE1 (PAI-1), a phylogenetically distinct but regulated coagulation factor is induced in the TM when treated with TGFβ2 which is proposed to inhibit ECM degradation via MMP regulation. TGFβ2 induced PAI-1 increased substrate elasticity and contribute aberrant ECM deposition in glaucoma^41^. However, the role of SERPINB2 and SERPINB11in the TM is not clearly understood. A detailed investigation in this line may give insight into the involvement of SERPINB2 and other members of this family in the pathogenesis of GC-induced glaucoma.

Similarly, neuropeptide (NPY) was significantly down-regulated only in 7 d treatment group (log2FC: − 6.7) and not found in 16 h post treatment. This peptide is known to be involved in various physiological and homeostatic processes in both central and peripheral nervous systems. The expression of NPY was documented in the retina of both mammalian and non-mammalian species where it is reported to prevent neuronal cell death induced by excitotoxic insults^42,43^*.* It is also expressed in drainage angle of mammalian eyes^44^. However, the physiological role of NPY in trabecular meshwork cells is not clearly understood which warrants a detailed investigation to understand its role in GC-OHT and glaucoma.

In this study, several functional pathways were significantly altered in both the groups after DEX treatment. The significant down-regulation of WNT signaling was observed only in 7 d DEX treated HTM cells with decreased expression of WNT10B (logFC =  − 2.8), WNT10A (logFC =  − 2.8), WNT7B (logFC =  − 2.6), WNT2 (logFC =  − 3.5) and WNT4 (logFC =  − 2.0). The WNT signaling antagonist SFRP2 (logFC =  − 3.7) was also identified as down-regulated only in 7 d treatment group. These findings suggest that the prolonged exposure of DEX in HTM cells resulted in the inhibition of WNT signaling. Previously, it was shown that there is an existence of a cross-inhibition between the TGFβ/Smad and canonical Wnt pathways in the TM and such cross-inhibition was mediated by Smad4 and β-catenin^45^. In a metanalysis of human microarray DEX expression datasets and bovine RNA -seq these two pathways were shown to be in TM cells in response to DEX treatment^46^. Among the 11 pathways that were altered between human and bovine TM cells in response to DEX treatment, only cell adhesion and Wnt signaling pathways were found similar in the present study.

In a recent study, it is shown that GR and WNT signaling inhibit each other in the TM, and that activation of Wnt signaling could prevent the adverse effect of glucocorticoids in the eye^47^. In the above study, elevated levels of Dickkopf-related protein1 (Dkk1) which is a canonical Wnt signaling inhibitor was found in the aqueous humor and TM of glaucoma patients. Dkk1 enhanced GR signaling and knockdown of Dkk1 or Wnt signaling activators decreased GR signaling in human TM cells. In contrast, the inhibition of canonical Wnt signaling by β-catenin knockdown increased GC-induced up-regulation of ECM proteins. In addition, it has been demonstrated that adenovirus mediated Wn3a expression decreased GC-induced OHT in mouse eyes. These observations strongly suggest that Wnt activation may be of potential strategy for the future treatment for GC-induced OHT and glaucoma.

The Rap1 signaling pathway was also significantly up-regulated only in 7 d treatment group. Rap1 regulates diverse cellular functions including focal adhesions, cell junction formation and cell polarity. A recent study by Zhu et al.^48^ identified rottlerin as a potential therapeutic agent for POAG through bio-informatics analysis of microarray data sets derived from POAG TMs and found that rottlerin could reverse the glaucomatous phenotype in both in vitro and steroid-induced experimental mouse model. Target prediction identified that Rap1 signaling as a part of a potential pathway underlying the effects of rottlerin. However, the role of Rap1 signaling has not been well studied in glaucoma. Further investigation into the role of the Rap1 signaling pathway in glaucoma including steroid glaucoma is warranted.

Interestingly, the cytokine-cytokine receptor interaction pathway was significantly enriched only in 16 h group as compared to 7 d group. This is in agreement with the observation by Peng et al.^49^ that this was the pathway which was significantly enriched in the aqueous humor of patients with POAG and Sturge–Weber syndrome induced secondary glaucoma. Cytokine-cytokine receptor interactions may play a role in immune-pathogenesis of glaucoma.

The findings of the present study may have a clinical relevance that DEX implants are increasingly being used for retinal vascular diseases due to increase in prevalence in our aging society. Long-term use of these implants results in elevated IOP which represents a major clinical challenge. This study opens up the avenues to design a steroid response susceptibility test to screen patients at risk before initiating steroid therapy for any eye inflammatory conditions. Some molecular targets with the potential of developing into drug therapy for the management of steroid glaucoma and POAG were identified in the present study.

Our study has some limitations. The history of steroid responsiveness of the donor eyes used in the present study was not known. Adaptation of an alternative method to better screen the steroid responsiveness of the donor eyes may provide a more disease appropriate cohort for examining global gene expression and aid in potential novel target identification. Due to limited availability of human donor eyes, HTM cells derived from two different ethnicities and RNA seq experiments conducted at two different centers were utilized in the present study which may contribute to variations in the gene expression profile. However, utmost care was taken to avoid the experimental variations by including appropriate vehicle controls for each DEX-treated HTM cells from the same donor.

In conclusion, this is the first report on the time-dependent effect of DEX on gene expression of primary human TM cells using RNA seq technology. Interestingly, both 16 h and 7 d DEX treatment groups demonstrated a distinct gene signature and their associated pathways in addition to common genes and pathways that were reported previously. Longer exposure of DEX in HTM cells showed down-regulation of Wnt signaling and up-regulation of Rap1 signaling. The cross-talk of these pathways with GR signaling warrants further experimental validation. Manipulation of these pathways have the potential to develop novel therapies for the management of GC-OHT/glaucoma.

## Materials and methods

### Human trabecular meshwork (HTM) cell culture

Human cadaveric eyes used for 16 h and 7 d duration were provided by the Liverpool Research Eye Bank, University of Liverpool, United Kingdom and Rotary Aravind International Eye Bank, Aravind Eye Hospital, Madurai, India, respectively. The characteristics of donor eyes used for this study are given in Supplementary Table S1. All eyes were examined under the dissecting microscope for any gross ocular pathological changes and only macroscopically normal eyes were used for the experiments. In Liverpool the medical and surgical history was available and all donors had no previous eye surgery or glaucoma. For eyes used in 7 d group, no medical and ocular history were available; however, the status of phakic/aphakic and history and duration of diabetes were available for those eye. Sixteen hours (16 h) (short DEX exposure time) and 7 days (7 d) long DEX exposure time) experiments were conducted at the University of Liverpool, UK and Aravind Medical Research Foundation, India, respectively.

Primary cultures of HTM cells were established and characterized as described previously^50–53^. Confluent cultures of HTM cells were treated with either 100 nM DEX or 0.1% ethanol (ETH) as a vehicle control for 16 h (n = 6) and 7 d (n = 8). The medium was exchanged every other day for 7 d treatment group. At the end of the respective treatments, RNA was extracted for gene expression profiling by RNA sequencing. Human TM cells between passages 3–7 were used for the experiments.

### RNA extraction

Total RNA was isolated from cultured TM cells after 16 h or 7 d of treatment using the commercial Qiagen Universal All Prep (Qiagen, UK) kit and TRIZOL reagent (Sigma, MO, USA) as per manufacturer’s specifications respectively. Total RNA was quantified initially on the Nanodrop-1000 (Thermofisher Scientific, DE, UK) and quality was determined by the Bioanalyser 2100 (Agilent, UK). HTM cells with an RIN > 9.5 and a concentration of 100 ng/μl or higher were used for RNA sequencing^54^.

### Expression profiling by RNA sequencing

RNA sequencing for 16 h and 7 d DEX–treated HTM cells were conducted at Exiqon Services, Denmark and Sandoor Life sciences, Hyderabad, India respectively as described previously^15,23^.

### RNA seq data analysis

The paired-end raw reads of both 16 h and 7 d DEX-treated HTM cells strains were subjected to bioinformatic analysis to identify differentially expressed genes (DEG). Briefly, the quality of each base was assessed by FastQC (http://www.bioinformatics.babraham.ac.uk/projects/fastqc/), and low-quality reads, PCR duplicates and adapter sequences were eliminated using Cutadapt3. The processed high quality paired end reads from each HTM cells were mapped with human reference genome hg38/GRCh38 using HISAT2^55^ and then expression of genes in read counts was obtained by quantification using FeatureCounts^56^. Genes with less than 10 read counts were excluded before global level normalization by Quintile strategy.

Differential gene expression analysis using raw counts was carried out using EdgeR from Bioconductor package in R programme^57^, with the DEX treated group compared to vehicle treated group at the 16 h and 7 d time points. Models of differential expression at a series of group comparisons were constructed using the limma package and a Benjamini–Hochberg (BH) correction was applied. Genes with absolute fold change (log2) value 2, and BH P value ≤ 0.05 were considered as DEGs and taken for further analysis. For comparison, the DEGs were segregated into five groups: Group A: DEGs between DEX and ETH treated for short duration (16 h); Group B: DEGs between DEX and ETH treated for longer duration (7 d); Group C: DEGs overlapping between Group A and Group B; Group D: Uniquely expressed DEGs of HTM cells exposed for 16 h (Group A minus Group C); Group E: Uniquely expressed DEGs of HTM cells exposed for 7 d (Group B minus Group C).

### Pathway enrichment analysis

Pathways associated with DEGs identified from RNA-seq were identified by Database for Annotation, Visualization and Integrated Discovery (DAVID) v6.8 (10.1186/gb-2003-4-9-r60)^58,59^. Pathways with significant P value (less than 0.05) and gene count greater than three were considered).

### Validation of RNA seq by qPCR

DEGs identified in the RNA seq data of 16 h and 7 d treatment groups were further validated by qPCR. Nine candidate genes were selected for q-PCR analysis based on their statistical significance in the RNA-seq data set and on the basis of published literature providing evidence of their involvement in glaucoma pathophysiology^12,15,17,20,23,27,40,51^. The list of primers for the selected genes for validation by qPCR is shown in the Supplementary Table S2. The expression of significantly altered differentially expressed genes identified in 16 h RNA seq data was validated by qPCR as described previously^51^. For 7 d treatment group, qPCR was performed using ABI-QuantStudio 5 (Applied Biosystems, MA, USA).

All mRNA were measured at CT threshold levels and normalized with the average CT values of a reference gene; GAPDH (for 16 h treatment). Values were expressed as fold increase over the corresponding values for control by the 2^−ΔΔCT^ method. The expression of genes in DEX treated HTM cells in logFC ratio was calculated by normalizing with reference control (ACTB; 7 d treatment) and vehicle control.

### Statistical analysis

All statistical analysis of expression of genes were carried out by using Graph Pad Prism (version.8.0.2) (Graph Pad software, CA, USA). Statistical significance between groups was analyzed by using un-paired two-tailed Student-t-test. All data are represented in mean ± Standard Error Mean (SEM) or otherwise specified. P value < 0.05 was considered as statistically significant.

### Institutional review board statement

This study was approved by the standing Human Ethics Committee of Aravind Medical Research Foundation, Madurai, Tamil Nadu, India (ID NO. RES2017006BAS-7 d treatment group) and University of Liverpool ethics review board (RETH000833-16 h treatment group) handled in accordance to the tenets of the Declaration of Helsinki.

## Supplementary Information

Supplementary Tables.

## Data Availability

The RNA-sequencing data of the present study are publicly available in NCBI (http://www.ncbi.nlm.nih.gov/sra) under the accession numbers: PRJNA781014 (16 h) and PRJNA729873 (7 d).
