# Author Correction: Short and long-term effect of dexamethasone on the transcriptome profile of primary human trabecular meshwork cells in vitro

**DOI:** 10.1038/s41598-022-17427-1

**Published:** 2022-07-28

**Authors:** Kandasamy Kathirvel, Karen Lester, Ravinarayanan Haribalaganesh, Ramasamy Krishnadas, Veerappan Muthukkaruppan, Brian Lane, David A. Simpson, Kasia Goljanek-Whysall, Carl Sheridan, Devarajan Bharanidharan, Colin E. Willoughby, Srinivasan Senthilkumari

**Affiliations:** 1 Department of Ocular Pharmacology, Aravind Medical Research Foundation, #1, Anna Nagar, Madurai, Tamil Nadu 625020 India; 2 Genomic Medicine, Biomedical Sciences Research Institute, Ulster University, Coleraine, Northern Ireland, UK; 3 Glaucoma Clinic, Aravind Eye Hospital, Madurai, Tamil Nadu India; 4 Aravind Medical Research Foundation, Madurai, Tamil Nadu India; 5 Translational Radiobiology Group, Division of Cancer Sciences, Manchester Academic Health Science Centre, Christie NHS Foundation Trust Hospital, University of Manchester, Manchester, M20 4BX UK; 6 Institute of Life Course and Medical Sciences, University of Liverpool, Liverpool, L7 8TX UK; 7 School of Medicine, Dentistry and Biomedical Sciences, The Wellcome – Wolfson Institute for Experimental Medicine, Queen’s University Belfast, Belfast, UK; 8 School of Medicine, Physiology, National University of Ireland Galway, Galway, H91 W5P7 Ireland; 9 Department of Eye and Vision Science, Institute of Life Course and Medical Sciences, University of Liverpool, Liverpool, L7 8TX UK; 10 Department of Bioinformatics, Aravind Medical Research Foundation, Madurai, Tamil Nadu India

Correction to: *Scientific Reports* 10.1038/s41598-022-12443-7, published online 18 May 2022

The original version of this Article contained an error in the spelling of the author Karen Lester, which was incorrectly given as Lester Karen.

The Author contribution statement now reads:

“Conceptualization: S.S. & C.E.W. Data Curation: S.S., C.E.W. & D.B. Formal Analysis: K.K., K.L., R.H., S.S., C.E.W. & D.B. Investigation: S.S., C.E.W. & D.B. Methodology: K.K., K.L. & R.H. Project administration: S.S. & C.E.W. Resources: S.S., C.E.W., D.B., D.A.S. Supervision: S.S. & C.E.W. Validation: S.S. & C.E.W. Software: D.B., K.K., K.L. Writing—Original Draft: K.K., K.L., R.H., S.S. & C.E.W. Writing—Review and Editing: D.B., R.K., V.M., B.L., D.A.S., K.G. & C.S. Funding acquisition: S.S., C.S. & C.E.W.”

The original Article and its accompanying Supplementary Information file have been corrected.

