# Supplementary material for: Short and long-term effect of dexamethasone on the transcriptome profile of primary human trabecular meshwork cells in vitro
Source: Sci Rep. 2022 May 18;12:8299. doi: 10.1038/s41598-022-12443-7 (PMC9117214; doi:10.1038/s41598-022-12443-7)
Supplement: Supplementary file 1 — Supplementary Tables. [file 41598_2022_12443_MOESM1_ESM.pdf]

Short and Long-Term Effect of Dexamethasone on the Transcriptome Profile of Primary Human Trabecular Meshwork Cells *In vitro*

[1,9,a]Kandasamy Kathirvel, [2,a] Karen Lester, [1]Ravinarayanan Haribalaganesh, [3]Ramasamy Krishnadas, [4]Veerappan Muthukkaruppan, [5,8]Brian Lane, [6]David A Simpson, [7,8]Kasia Goljanek-Whysall, [8]Carl Sheridan, [9]Devarajan Bharanidharan, [2,8]Colin E. Willoughby*, [1]Srinivasan Senthilkumari*

[1]Department of Ocular Pharmacology, Aravind Medical Research Foundation, Madurai, Tamilnadu, India.
[2]Genomic Medicine, Biomedical Sciences Research Institute, Ulster University, Northern Ireland, United Kingdom.
[3]Glaucoma Clinic, Aravind Eye Hospital, Madurai, Tamilnadu, India.
[4]Advisor, Aravind Medical Research Foundation, Madurai, Tamilnadu, India.
[5]Translational Radiobiology Group, Division of Cancer Sciences, University of Manchester, Manchester Academic Health Science Centre, Christie NHS Foundation Trust Hospital, Manchester, M20 4BX, United Kingdom. Institute of Life Course and Medical Sciences, University of Liverpool, Liverpool, L7 8TX, United Kingdom.
[6]The Wellcome – Wolfson Institute for Experimental Medicine, School of Medicine, Dentistry and Biomedical Sciences, Queen’s University Belfast, United Kingdom.
[7]School of Medicine, Physiology, National University of Ireland Galway, Galway, H91 W5P7, Ireland.
[8] Department of Eye and Vision Science, Institute of Life Course and Medical Sciences, University of Liverpool, Liverpool, L7 8TX, United Kingdom.
[9]Department of Bioinformatics, Aravind Medical Research Foundation, Madurai, Tamilnadu, India.

***Corresponding Authors:**

Srinivasan Senthilkumari, M. Pharm, Ph. D

Department of Ocular Pharmacology, Aravind Medical Research Foundation

#1, Anna Nagar, Madurai-625020, Tamilnadu, India

Tele (0): +91-452-4356550; extn. 438; Fax: +91-452-2530984;

e.mail: ss_kumari@aravind.org

Prof. Colin E. Willoughby

Genomic Medicine, Biomedical Sciences Research Institute,

Ulster University, Northern Ireland, United Kingdom
e.mail: c.willoughby@ulster.ac.uk

[a] Contributed equally as first authors

Table S1: Characteristics of Human Donor Eyes Used for the Present Study

DEX Treatment (nM)	Duration	Code	Age	Sex	Treatment
100nM (N=6)	16 h	LGP-1	57	M	ETH/DEX
		LGP-2	65	M	ETH/DEX
		NTM002	57	M	ETH/DEX
		NTM009	57	M	ETH/DEX
		NTM720	64	M	ETH/DEX
		NTM119	94	M	ETH/DEX
100nM (N=8)	7 d	OCHD18-39	82	F	ETH/DEX
		OCHD18-49	48	M	ETH/DEX
		OCHD18-52	55	F	ETH/DEX

		OCHD18-53	67	M	ETH/DEX
		OCHD18-56	82	M	ETH/DEX
		OCHD19-02	66	M	ETH/DEX
		OCHD19-03	65	F	ETH/DEX
		OCHD19-04	72	M	ETH/DEX

Table S2: Primer Pairs for RNA-Seq Validation (16 h)

S. No	Gene Target	Primer Sequence	
		16 hours	7 days
1	FKBP5	F:5’CTCCCTAAAATTCCCTCGAATGC’3	F:5'-AAGGCCAAGTCATCAAGGCA-3'
		R:5’CCCTCTCCTTTCCGTTTGGTT’3	R:5'-TGAAATCAAGGAGCTCAATCTC-3'
2	FST	F:5’AGGCAAGATGTAAAGAGCAGC’3	F:5'-GCTGTGCCCTGACAGTAAGT-3'
		R:5’CAGTAGGCATTATTGGTCTGGTC’3	R:5'-GTGTCTTCCGAAATGGAGTTGC-3'
3	MMP1	F: 5’ACGGATACCCCAAGGACATCT’3	F:5'- ACGGATACCCCAAGGACATCT-3'
		R: 5’TCAGAAAGAGCATCGATATG’3	R:5'-TCAGAAAGAGCAGCATCGATATG-3'
4	PDPN	F: 5’AACCAGCGAAGACCGCTATAA’3	F:5'-AACCAGCGAAGACCGCTATAA-3'
		R: 5’CGAATGCCTGTTACACTGTTGA’3	R:5'-CGAATGCCTGTTACACTGTTGA-3'
5	PTK2B	F: 5’CCCCTGAGTCGAGTAAAGTTGG’3	F:5'-CCCCTGAGTCGAGTAAAGTTGG-3'
		R: 5’GATACGCACGTCCTCCTTTTC’3	R:5'-GATACGCACGTCCTCCTTTTC-3'
6	RGCC	F: 5’CGCCACTTCCACTACGAGG’3	F:5'-CGCCACTTCCACTACGAGG-3'
		R: 5’CAGCAATGAAGGCTTCTAGCTC’3	R:5'-CAGCAATGAAGGCTTCTAGCTC-3'
7	SERPINB2	F: 5’TCCTGGGTCAAGACTCAAACC’3	F:5'-TCCTGGGTCAAGACTCAAACC-3'
		R: 5’CATCCTGGTATCCCCATCTACAG’3	R:5'-CATCCTGGTATCCCCATCTACAG-3'
8	VEGFC	F: 5’GAGGAGCAGTTACGGTCTGTG’3	F:5'-GAGGAGCAGTTACGGTCTGTG-3'
		R: 5’TCCTTTCCTTAGCTGACACTTGT’3	R:5'-TCCTTTCCTTAGCTGACACTTGT-3'
9	ZBTB16	F: 5’CCTCAGACGACAATGACACGG’3	F:5'-CCTCAGACGACAATGACACGG-3'
		R: 5’CTCGCTGGAATGCTTCGAGAT’3	R:5'-CTCGCTGGAATGCTTCGAGAT-3'

Table S3a: List of top 50 Up/Down-regulated Genes from Group A

Up-regulated genes				Down-regulated genes			
**Gene**	**logFC**	**logCPM**	**P Value**	**Gene**	**logFC**	**logCPM**	**P Value**
ZBTB16	7.6991	2.144367	3.93E-06	TP63	-4.75466	-1.9076	0.003015
OCA2	6.817079	2.019772	2.63E-10	KRTAP4-7	-4.64115	-2.75702	0.002146
LEP	6.212262	-1.32218	5.15E-04	GJB2	-4.36855	-2.86834	0.00134
CCAT1	5.913344	-2.70286	0.002979	TMEM108-AS1	-4.27057	-0.5583	0.001793
CIDEC	5.78612	-2.78275	0.001021	LRRN1	-4.14203	-3.63259	0.044332
NDST4	5.767665	-2.75183	0.010945	FGF16	-4.13737	-2.99608	0.016576
OLAH	5.744702	0.392163	3.05E-10	SPATA31E1	-4.11655	-3.07267	0.006086
CNR1	5.689774	0.87157	0.001218	SUCNR1	-4.09291	-3.04633	0.005503
FAM105A	5.647441	2.781821	3.79E-10	CCDC141	-4.08231	-3.05163	0.002129
SAA1	5.645963	1.380466	6.89E-09	SERPINB2	-3.73461	2.275158	6.92E-04
ALOX15B	5.638745	0.257532	1.95E-08	FAM19A3	-3.7216	-1.32076	4.21E-06
HIF3A	5.4497	-1.98756	4.43E-05	GABRA4	-3.69131	-1.92586	0.011211
LGI3	5.401532	-0.00197	1.32E-07	ELOVL2-AS1	-3.61024	-2.88914	0.013646
SAA2-SAA4	5.290503	-3.08574	8.83E-04	IVL	-3.55164	3.96E-04	0.003206
C7	5.023251	-3.22535	0.014352	INSM2	-3.54622	-2.64853	0.032325
UBA52P8	4.977612	-3.26546	0.00223	LCE3A	-3.52099	-2.26775	0.016712
PNMT	4.936361	-3.25641	0.003935	LCE1C	-3.49153	-3.31426	0.019634
CCL3L3	4.891576	-3.29353	0.012138	NTSR1	-3.47662	0.253475	0.008717
ALOX5AP	4.836676	-0.54226	2.57E-07	TGFA	-3.42263	0.170103	0.006218
FAM107A	4.811483	0.917285	1.42E-05	CXCR4	-3.41723	-1.98838	0.010154
RGCC	4.790965	3.861133	0.001081	KRT19	-3.40331	4.891026	1.92E-05
LINC00964	4.523061	-2.6008	0.001364	RNU6-118P	-3.39061	-3.38484	0.035994
ACKR1	4.504368	0.126657	0.021377	MS4A12	-3.36186	-3.42068	0.043333
CADM3-AS1	4.46674	-3.48871	0.034944	TNFRSF9	-3.31793	-2.97307	0.025971
OR3A1	4.426009	-3.48658	0.010772	NR1D1	-3.1518	2.813576	1.65E-12
H19	4.419902	0.243194	3.86E-04	SLC7A14	-3.14203	1.990144	1.45E-04
RBMY1A3P	4.373364	-3.52879	0.027555	FER1L6	-3.14159	-2.30399	0.010493
ANGPTL7	4.286227	1.043775	0.001021	MCHR1	-3.09605	0.077106	0.002953
APOB	4.261701	-1.74598	0.01705	CYTIP	-3.06451	-2.15903	0.014672
LINC00332	4.254916	-2.83157	0.012106	CSF2	-3.05834	-0.79702	0.00393
ZNF840	4.240351	-3.57816	0.032746	RNU6-137P	-2.9772	-3.26842	0.007867
PDK4	4.141157	2.437951	4.46E-07	CDC20B	-2.97361	-2.95319	0.017313
MOB3B	4.110102	0.242993	1.66E-05	KRT16	-2.89471	-0.55725	0.028857
SLC17A3	3.898265	-2.99778	0.009722	KRTAP4-12	-2.81493	-2.89381	0.019184
ADH1B	3.893459	1.189551	0.014387	TACR1	-2.74512	-1.82516	0.007374
SAA2	3.816066	-1.28468	6.66E-04	EVI2A	-2.72873	-1.2672	7.51E-04

OMD	3.759902	0.540711	1.32E-04	EBF3	-2.72184	-2.56649	0.03766
IP6K3	3.756283	1.007611	4.38E-07	GRPR	-2.66534	1.046002	4.37E-06
IL18	3.712121	-1.16629	0.001141	IL1B	-2.62255	2.070758	1.37E-04
LIX1	3.620558	-2.37076	0.014003	CSNK1A1P1	-2.61533	-2.28419	0.024971
MARCH10	3.612298	-0.59562	1.25E-04	ESM1	-2.56555	6.962024	0.019522
CD163	3.570825	-1.86626	0.031175	GRIN2A	-2.54737	-0.36879	0.002351
KDF1	3.503209	-2.82669	0.029132	TRIML2	-2.54023	-1.33735	0.010246
CDH20	3.485157	2.805804	2.95E-07	BCL2A1	-2.52814	-1.33764	0.0081
C10orf10	3.451466	4.12064	2.47E-04	IL1RN	-2.51691	-0.78863	0.019984
NKD1	3.450642	-1.5778	0.004903	ERMN	-2.49034	-0.56562	0.002515
MAOA	3.445347	5.513373	2.42E-13	PTHLH	-2.4542	1.947604	0.002709
WISP1	3.442528	2.116349	0.002748	FST	-2.45314	7.46288	1.43E-05
KLF15	3.415696	0.338311	5.41E-04	ALDH1A3	-2.4271	5.95997	2.56E-09
STK31	3.386502	-2.10787	0.012172	KCNK3	-2.42547	0.497296	0.04803

Note: *Group A:* DEGs between DEX and ETH treated for short duration (16h)

Table S3b: List of top 50 Up/Down-regulated Genes from Group B.

Up-regulated genes				Down-regulated genes			
**Gene**	**logFC**	**logCPM**	**P Value**	**Gene**	**logFC**	**logCPM**	**P Value**
ZBTB16	6.469771	4.645611	2.79E-22	NPY	-6.70151	2.337886	2.66E-04
SAA1	6.219889	2.214826	9.59E-11	PRSS22	-6.38518	-1.47254	8.02E-04
MYOC	5.786683	8.501142	6.47E-05	LRRC26	-6.30496	0.500688	0.001154
OCA2	5.710359	1.977641	5.91E-12	HOXB13	-6.26252	1.925207	0.001245
H19	5.52922	8.142276	6.91E-11	ACPP	-6.24787	5.740014	1.63E-04
BHLHE22	4.795929	-0.16536	0.005898	RLN1	-6.19291	0.229894	0.001526
APOD	4.740998	8.023247	1.18E-04	KLK3	-6.19217	6.494438	3.79E-04
SAA2	4.655101	-0.07569	1.30E-04	PRAC1	-6.081	-1.02086	0.002144
HIF3A	4.598946	3.556768	4.05E-07	GIMAP1	-6.03295	-1.72779	1.51E-04
PRODH	4.54025	2.918714	3.66E-11	KLK2	-6.00607	5.321405	8.81E-04
FKBP5	4.467148	7.460413	3.19E-23	KLK4	-5.94843	2.034524	0.002213
P2RY14	4.355578	1.076507	6.20E-08	AQP1	-5.90064	6.706375	6.83E-09
LSP1	4.283072	5.109946	4.60E-09	MSMB	-5.77753	2.981128	0.002492
SCN3A	4.248788	2.547229	7.88E-05	ST14	-5.77001	0.500316	7.21E-04
ANGPTL7	4.233551	9.075265	0.001147	PAGE4	-5.67683	-0.50939	0.003169
PRR33	4.182477	0.924015	3.21E-08	TPSAB1	-5.66112	-0.27503	7.08E-04
CPM	4.017027	3.646993	1.90E-07	TPSB2	-5.64276	-0.02604	0.001383
LEP	3.854555	-0.92605	0.002286	KRT15	-5.59983	2.359236	7.93E-05
IGF2	3.777841	6.594018	2.10E-09	LINC01297	-5.42824	0.161739	0.001658
IGF2-AS	3.771141	-1.96988	3.91E-04	HOXA10	-5.36139	-0.80501	3.15E-04
ADH1B	3.707569	7.121667	7.38E-06	GIMAP7	-5.35117	-1.16462	0.003777
RGCC	3.592186	1.824843	7.12E-04	IGHG3	-5.30586	1.084123	0.009352
LINC00702	3.565781	4.414582	8.68E-08	HOXA9	-5.2873	-1.6163	0.005055
SLC16A12	3.545497	0.904799	2.16E-06	HLA-DQB1	-5.27142	0.069513	0.003274
ANGPTL1	3.539448	3.075954	4.72E-08	KRT5	-5.2671	1.442267	3.34E-04
SAMHD1	3.46755	7.654626	4.37E-14	UPK3A	-5.23658	-0.57963	0.009173
FHL5	3.453078	-0.55363	0.001374	AZGP1	-5.23442	3.090287	0.003695
LINC01088	3.439059	1.030156	2.67E-06	IGLC3	-5.23234	0.617578	0.005658
STEAP4	3.272005	2.873029	4.60E-04	RAMP3	-5.22737	-0.65501	0.001978
MAOA	3.219507	4.53732	7.03E-05	COL9A1	-5.18985	1.745759	2.42E-05
TRAV39	3.184529	-1.04837	0.002723	DES	-5.18691	4.97087	5.67E-05
FMO2	3.162058	2.670166	4.63E-05	MT1H	-5.17696	-2.24801	0.003385
ANKRD2	3.10094	0.494111	6.55E-04	FXYD3	-5.16764	1.00437	6.76E-04
CHI3L2	3.019697	3.101092	0.003799	CCDC64B	-5.16744	-0.4844	6.07E-04
STOX1	2.978317	1.660544	0.005573	MMP7	-5.16594	0.817164	6.99E-04
KCNE1	2.952832	0.259925	9.97E-04	CHRNA2	-5.16025	0.306457	0.001386

GIP	2.952648	-1.75823	8.43E-04	GYLTL1B	-5.0962	-0.77806	8.74E-04
MRO	2.927396	1.824722	0.001177	PLVAP	-5.06443	0.634955	5.30E-04
MIR5690	2.9135	-1.95506	0.0026	SPDEF	-5.00996	1.499005	8.58E-04
TLDC2	2.904924	1.184351	1.31E-06	CD177	-4.98374	0.275334	0.002824
ADRA1B	2.895918	2.880945	2.01E-09	SERPINB11	-4.96923	-0.10361	0.002876
ADH1A	2.88262	1.394164	3.12E-04	DIO3OS	-4.93426	-1.17936	0.007826
NTRK2	2.871932	5.180909	0.003317	SFN	-4.93102	0.825148	7.12E-05
XRCC6P2	2.858951	-0.32354	2.88E-05	KCNQ1	-4.9203	-1.8196	0.007267
NEDD9	2.790817	7.654906	1.77E-05	HOXA13	-4.91558	0.125708	4.15E-04
USP2	2.754429	2.387434	9.92E-05	FAM110D	-4.90182	-1.43618	0.006624
LINC00525	2.716197	-2.25042	0.003534	IRX4	-4.89999	-1.83535	0.004901
SLC16A10	2.714817	0.999613	0.001783	HLA-DRA	-4.88604	1.815739	0.002646
NRCAM	2.694034	4.941819	0.004455	C1orf116	-4.87612	0.877927	2.02E-04
FAS-AS1	2.693421	-2.50119	0.011674	PDE9A	-4.84261	0.385632	2.25E-05

Note: *Group B:* DEGs between DEX and ETH treated for longer duration (7d)

Table S3c: List of overlapping genes from Group C

	Group A			Group B		
**Gene**	**logFC**	**logCPM**	**P Value**	**logFC**	**logCPM**	**P Value**
ZBTB16	7.6991	2.144367	3.93E-06	6.469771	4.645611	2.79E-22
OCA2	6.817079	2.019772	2.63E-10	5.710359	1.977641	5.91E-12
LEP	6.212262	-1.32218	5.15E-04	3.854555	-0.92605	0.002286
OLAH	5.744702	0.392163	3.05E-10	2.494219	1.630462	3.80E-04
SAA1	5.645963	1.380466	6.89E-09	6.219889	2.214826	9.59E-11
HIF3A	5.4497	-1.98756	4.43E-05	4.598946	3.556768	4.05E-07
LGI3	5.401532	-0.00197	1.32E-07	2.023395	-0.28967	0.023957
C7	5.023251	-3.22535	0.014352	-2.47034	1.612644	0.041657
RGCC	4.790965	3.861133	0.001081	3.592186	1.824843	7.12E-04
H19	4.419902	0.243194	3.86E-04	5.52922	8.142276	6.91E-11
ANGPTL7	4.286227	1.043775	0.001021	4.233551	9.075265	0.001147
PDK4	4.141157	2.437951	4.46E-07	2.600955	5.120345	5.04E-06
MOB3B	4.110102	0.242993	1.66E-05	2.535956	2.25653	2.67E-04
ADH1B	3.893459	1.189551	0.014387	3.707569	7.121667	7.38E-06
SAA2	3.816066	-1.28468	6.66E-04	4.655101	-0.07569	1.30E-04
MARCH10	3.612298	-0.59562	1.25E-04	2.047767	-0.92857	0.022404
MAOA	3.445347	5.513373	2.42E-13	3.219507	4.53732	7.03E-05
KLF15	3.415696	0.338311	5.41E-04	2.285	4.633049	1.13E-10
CPM	3.084205	3.505109	0.001022	4.017027	3.646993	1.90E-07
MRO	2.928403	1.525505	0.021691	2.927396	1.824722	0.001177
SLCO2A1	2.918312	-1.4591	0.033547	-4.67337	0.103155	5.64E-04
FKBP5	2.910129	7.361695	2.02E-10	4.467148	7.460413	3.19E-23
RASSF6	2.846302	-1.81284	0.003284	-2.43413	-2.057	0.046611
ITGA10	2.731467	3.131551	1.34E-05	2.067832	5.009811	1.16E-04
SLC16A12	2.599798	1.421226	0.00106	3.545497	0.904799	2.16E-06
GIP	2.442996	-1.55192	0.002565	2.952648	-1.75823	8.43E-04
ANGPTL1	2.344392	2.343466	0.01012	3.539448	3.075954	4.72E-08
PLCE1-AS1	2.336548	3.074643	4.05E-04	2.278802	4.093517	1.11E-04
NRCAM	2.294725	5.445473	1.89E-06	2.694034	4.941819	0.004455
AOX1	2.269174	7.197862	2.36E-11	2.469175	6.282018	6.44E-05
POM121L9P	2.264301	1.755788	0.002727	2.205378	2.983997	0.001329
LINC01088	2.133096	-2.71421	0.044759	3.439059	1.030156	2.67E-06
PER1	2.116311	4.298915	8.99E-11	2.165042	6.005285	1.58E-15
TSC22D3	2.059884	5.637636	1.02E-07	2.092664	8.142146	1.52E-10
KRT15	-2.04715	0.28964	4.90E-04	-5.59983	2.359236	7.93E-05
GAP43	-2.05724	0.308887	0.027793	-2.02486	2.298992	0.002303

EHF	-2.1198	-0.59468	0.002435		-2.95552	-0.53237	0.017395
ELOVL2	-2.19882	2.970598	0.038038		-2.00457	2.755107	1.36E-04
ROBO4	-2.22779	-2.50759	0.020966		-2.74161	-0.83303	9.41E-04
PKP1	-2.31957	-0.4564	0.044056		-3.77574	1.054377	1.35E-04
KCNN3	-2.3687	-2.6713	0.010176		-2.05838	-0.86625	3.79E-04
FST	-2.45314	7.46288	1.43E-05		-2.04443	6.592413	1.18E-10
MCHR1	-3.09605	0.077106	0.002953		-2.71202	-0.3586	0.00281
SLC7A14	-3.14203	1.990144	1.45E-04		-2.11287	1.399396	0.002158
FAM19A3	-3.7216	-1.32076	4.21E-06		-3.27296	-1.49499	5.89E-04
GJB2	-4.36855	-2.86834	0.00134		-3.02598	-1.53398	0.006085
TP63	-4.75466	-1.9076	0.003015		-2.41187	-0.57334	0.006497

Note: *Group C:* DEGs that overlapping between Group A and Group B

Table S3d: List of top 50 Up/Down-regulated Genes from Group D

Up-regulated genes				Down-regulated genes			
**Gene**	**logFC**	**logCPM**	**P Value**	**Gene**	**logFC**	**logCPM**	**P Value**
CCAT1	5.913344	-2.70286	0.002979	KRTAP4-7	-4.64115	-2.75702	0.002146
CIDEC	5.78612	-2.78275	0.001021	TMEM108-AS1	-4.27057	-0.5583	0.001793
NDST4	5.767665	-2.75183	0.010945	LRRN1	-4.14203	-3.63259	0.044332
CNR1	5.689774	0.87157	0.001218	FGF16	-4.13737	-2.99608	0.016576
FAM105A	5.647441	2.781821	3.79E-10	SPATA31E1	-4.11655	-3.07267	0.006086
ALOX15B	5.638745	0.257532	1.95E-08	SUCNR1	-4.09291	-3.04633	0.005503
SAA2-SAA4	5.290503	-3.08574	8.83E-04	CCDC141	-4.08231	-3.05163	0.002129
UBA52P8	4.977612	-3.26546	0.00223	SERPINB2	-3.73461	2.275158	6.92E-04
PNMT	4.936361	-3.25641	0.003935	GABRA4	-3.69131	-1.92586	0.011211
CCL3L3	4.891576	-3.29353	0.012138	ELOVL2-AS1	-3.61024	-2.88914	0.013646
ALOX5AP	4.836676	-0.54226	2.57E-07	IVL	-3.55164	3.96E-04	0.003206
FAM107A	4.811483	0.917285	1.42E-05	INSM2	-3.54622	-2.64853	0.032325
LINC00964	4.523061	-2.6008	0.001364	LCE3A	-3.52099	-2.26775	0.016712
ACKR1	4.504368	0.126657	0.021377	LCE1C	-3.49153	-3.31426	0.019634
CADM3-AS1	4.46674	-3.48871	0.034944	NTSR1	-3.47662	0.253475	0.008717
OR3A1	4.426009	-3.48658	0.010772	TGFA	-3.42263	0.170103	0.006218
RBMY1A3P	4.373364	-3.52879	0.027555	CXCR4	-3.41723	-1.98838	0.010154
APOB	4.261701	-1.74598	0.01705	KRT19	-3.40331	4.891026	1.92E-05
LINC00332	4.254916	-2.83157	0.012106	RNU6-118P	-3.39061	-3.38484	0.035994
ZNF840	4.240351	-3.57816	0.032746	MS4A12	-3.36186	-3.42068	0.043333
SLC17A3	3.898265	-2.99778	0.009722	TNFRSF9	-3.31793	-2.97307	0.025971
OMD	3.759902	0.540711	1.32E-04	NR1D1	-3.1518	2.813576	1.65E-12
IP6K3	3.756283	1.007611	4.38E-07	FER1L6	-3.14159	-2.30399	0.010493
IL18	3.712121	-1.16629	0.001141	CYTIP	-3.06451	-2.15903	0.014672
LIX1	3.620558	-2.37076	0.014003	CSF2	-3.05834	-0.79702	0.00393
CD163	3.570825	-1.86626	0.031175	RNU6-137P	-2.9772	-3.26842	0.007867
KDF1	3.503209	-2.82669	0.029132	CDC20B	-2.97361	-2.95319	0.017313
CDH20	3.485157	2.805804	2.95E-07	KRT16	-2.89471	-0.55725	0.028857
C10orf10	3.451466	4.12064	2.47E-04	KRTAP4-12	-2.81493	-2.89381	0.019184
NKD1	3.450642	-1.5778	0.004903	TACR1	-2.74512	-1.82516	0.007374
WISP1	3.442528	2.116349	0.002748	EVI2A	-2.72873	-1.2672	7.51E-04
STK31	3.386502	-2.10787	0.012172	EBF3	-2.72184	-2.56649	0.03766
GDF7	3.382018	-3.30062	0.034976	GRPR	-2.66534	1.046002	4.37E-06
HLF	3.360261	-2.63573	0.031377	IL1B	-2.62255	2.070758	1.37E-04
SNORA9	3.355249	-2.88268	0.001048	CSNK1A1P1	-2.61533	-2.28419	0.024971
GPR64	3.269424	0.864129	7.78E-05	ESM1	-2.56555	6.962024	0.019522

PPP1R1B	3.250992	-2.90152	0.025118	GRIN2A	-2.54737	-0.36879	0.002351
SERF1A	3.246102	-3.34594	0.039961	TRIML2	-2.54023	-1.33735	0.010246
IL1R2	3.200496	-2.58345	0.005751	BCL2A1	-2.52814	-1.33764	0.0081
SORBS2	3.186627	4.722545	1.09E-09	IL1RN	-2.51691	-0.78863	0.019984
IFNLR1	3.176104	-2.9785	0.024329	ERMN	-2.49034	-0.56562	0.002515
TPRG1-AS1	3.147698	-2.65822	0.002045	PTHLH	-2.4542	1.947604	0.002709
NEBL	3.091387	0.896332	0.001423	ALDH1A3	-2.4271	5.95997	2.56E-09
EFHD1	3.085808	0.75279	0.012593	KCNK3	-2.42547	0.497296	0.04803
ANKRD1	3.072437	4.902271	5.86E-05	NR1D2	-2.42076	5.240801	9.88E-15
PRR15	3.045917	-1.13035	0.005165	CDCP1	-2.39784	5.178189	4.04E-07
FOXO1	2.997491	3.322099	1.43E-04	IFNWP2	-2.3244	-2.68881	0.046495
GRIA1	2.995596	-0.06457	0.036469	USP50	-2.31103	-2.9698	0.028022
LRRTM3	2.870623	-1.40713	0.010561	NRG1	-2.30869	4.163404	3.49E-06
LMO3	2.78625	1.694504	0.004888	MIR146A	-2.28566	1.09297	0.00439

Note: *Group D:* Uniquely expressed DEGs of HTM cells exposed for 16h (Group A minus Group C)

Table S3e: List of top 50 Up/Down-regulated Genes from Group E

Up-regulated genes				Down-regulated genes			
**Gene**	**logFC**	**logCPM**	**P Value**	**Gene**	**logFC**	**logCPM**	**P Value**
MYOC	5.786683	8.501142	6.47E-05	NPY	-6.70151	2.337886	2.66E-04
BHLHE22	4.795929	-0.16536	0.005898	PRSS22	-6.38518	-1.47254	8.02E-04
APOD	4.740998	8.023247	1.18E-04	LRRC26	-6.30496	0.500688	0.001154
PRODH	4.54025	2.918714	3.66E-11	HOXB13	-6.26252	1.925207	0.001245
P2RY14	4.355578	1.076507	6.20E-08	ACPP	-6.24787	5.740014	1.63E-04
LSP1	4.283072	5.109946	4.60E-09	RLN1	-6.19291	0.229894	0.001526
SCN3A	4.248788	2.547229	7.88E-05	KLK3	-6.19217	6.494438	3.79E-04
PRR33	4.182477	0.924015	3.21E-08	PRAC1	-6.081	-1.02086	0.002144
IGF2	3.777841	6.594018	2.10E-09	GIMAP1	-6.03295	-1.72779	1.51E-04
IGF2-AS	3.771141	-1.96988	3.91E-04	KLK2	-6.00607	5.321405	8.81E-04
LINC00702	3.565781	4.414582	8.68E-08	KLK4	-5.94843	2.034524	0.002213
SAMHD1	3.46755	7.654626	4.37E-14	AQP1	-5.90064	6.706375	6.83E-09
FHL5	3.453078	-0.55363	0.001374	MSMB	-5.77753	2.981128	0.002492
STEAP4	3.272005	2.873029	4.60E-04	ST14	-5.77001	0.500316	7.21E-04
TRAV39	3.184529	-1.04837	0.002723	PAGE4	-5.67683	-0.50939	0.003169
FMO2	3.162058	2.670166	4.63E-05	TPSAB1	-5.66112	-0.27503	7.08E-04
ANKRD2	3.10094	0.494111	6.55E-04	TPSB2	-5.64276	-0.02604	0.001383
CHI3L2	3.019697	3.101092	0.003799	LINC01297	-5.42824	0.161739	0.001658
STOX1	2.978317	1.660544	0.005573	HOXA10	-5.36139	-0.80501	3.15E-04
KCNE1	2.952832	0.259925	9.97E-04	GIMAP7	-5.35117	-1.16462	0.003777
MIR5690	2.9135	-1.95506	0.0026	IGHG3	-5.30586	1.084123	0.009352
TLDC2	2.904924	1.184351	1.31E-06	HOXA9	-5.2873	-1.6163	0.005055
ADRA1B	2.895918	2.880945	2.01E-09	HLA-DQB1	-5.27142	0.069513	0.003274
ADH1A	2.88262	1.394164	3.12E-04	KRT5	-5.2671	1.442267	3.34E-04
NTRK2	2.871932	5.180909	0.003317	UPK3A	-5.23658	-0.57963	0.009173
XRCC6P2	2.858951	-0.32354	2.88E-05	AZGP1	-5.23442	3.090287	0.003695
NEDD9	2.790817	7.654906	1.77E-05	IGLC3	-5.23234	0.617578	0.005658
USP2	2.754429	2.387434	9.92E-05	RAMP3	-5.22737	-0.65501	0.001978
LINC00525	2.716197	-2.25042	0.003534	COL9A1	-5.18985	1.745759	2.42E-05
SLC16A10	2.714817	0.999613	0.001783	DES	-5.18691	4.97087	5.67E-05
FAS-AS1	2.693421	-2.50119	0.011674	MT1H	-5.17696	-2.24801	0.003385
SOAT2	2.679968	-1.23965	0.004303	FXYD3	-5.16764	1.00437	6.76E-04
MYBPHL	2.675798	-2.4836	0.017486	CCDC64B	-5.16744	-0.4844	6.07E-04
ADH4	2.6586	-1.13255	2.35E-04	MMP7	-5.16594	0.817164	6.99E-04
GALNT15	2.640343	4.553566	0.001341	CHRNA2	-5.16025	0.306457	0.001386
LINC00704	2.63911	-2.11364	0.005001	GYLTL1B	-5.0962	-0.77806	8.74E-04

FGFR4	2.629364	2.585506	0.001299	PLVAP	-5.06443	0.634955	5.30E-04
RNA5SP111	2.620581	-1.85238	0.02188	SPDEF	-5.00996	1.499005	8.58E-04
TNFAIP8L3	2.611999	2.800751	2.60E-05	CD177	-4.98374	0.275334	0.002824
TRPC3	2.600795	1.446691	0.001774	SERPINB11	-4.96923	-0.10361	0.002876
TIMP4	2.579974	1.921086	4.98E-05	DIO3OS	-4.93426	-1.17936	0.007826
FPR1	2.558859	0.451366	0.001327	SFN	-4.93102	0.825148	7.12E-05
ACA59	2.544022	-1.2974	4.81E-04	KCNQ1	-4.9203	-1.8196	0.007267
C3	2.519924	5.773602	0.011548	HOXA13	-4.91558	0.125708	4.15E-04
SLCO4A1	2.503841	0.894804	0.031207	FAM110D	-4.90182	-1.43618	0.006624
MAP1LC3C	2.500321	2.824507	0.013682	IRX4	-4.89999	-1.83535	0.004901
MIR5685	2.497169	-2.37282	0.006539	HLA-DRA	-4.88604	1.815739	0.002646
KIAA1456	2.462965	3.18646	0.003078	C1orf116	-4.87612	0.877927	2.02E-04
RAMP2-AS1	2.457799	0.756573	0.019992	PDE9A	-4.84261	0.385632	2.25E-05
FAM46B	2.384404	4.720006	3.44E-06	RAB25	-4.78721	-0.64192	9.59E-04

Note: *Group E:* Uniquely expressed DEGs of HTM cells exposed for 7d (Group B minus Group C)
